# Impact of parathyroid Hormone’s dose, timing, and delivery mode on osteoclastogenesis and osteogenesis using a human periodontal ligament fibroblast model

**DOI:** 10.3389/fphar.2025.1661759

**Published:** 2025-11-18

**Authors:** Wael M. I. Talbi, Elizabeth Steemers, Jolanda M. A. Hogervorst, Ton Schoenmaker, Teun J. de Vries

**Affiliations:** 1 Department of Periodontology, Academic Centre for Dentistry Amsterdam (ACTA), University of Amsterdam and Vrije Universiteit, Amsterdam, Netherlands; 2 Department of Oral Cell Biology, Academic Centre for Dentistry Amsterdam (ACTA), University of Amsterdam and Vrije Universiteit, Amsterdam, Netherlands

**Keywords:** parathyroid hormone, teriparatide, osteoclast, osteogenesis, periodontal ligamentfibroblast, co-culture model, bone regeneration, inflammatory signaling

## Abstract

Bone drug parathyroid hormone (PTH or teriparatide) has dual functions: it is a widely used anabolic drug resulting in increased bone formation, but it also activates osteoclasts. To further investigate the mechanistic insight of dose, timing, and delivery mode, a suitable cell model, periodontal ligament fibroblast (PDLF), was used to study PTH’s role in osteoclastogenesis and osteogenesis. PDLF were either co-cultured with peripheral blood mononuclear cells (PBMC) to study osteoclastogenesis or cultured under osteogenic conditions to study osteogenesis. A PTH titration (10^–12^ to 10^–9^ M) for 3 weeks identified the optimal PTH dose. Optimal PTH (10^–9^ M) dose was then administered under six regimens: continuous, first week only, second week only, third week only, and intermittent. At day 21, outcomes included osteoclast number and multinuclearity, gene expression (qPCR) for osteoclastogenesis and alkaline phosphatase (ALP) activity, and Alizarin Red staining for osteogenesis. PTH enhanced osteoclast formation in a dose-dependent manner (p = 0.008), with 10^–9^ M being most effective. Timing was critical: first- and second-week exposure significantly increased osteoclast number and size, while continuous or late exposure had minimal effect. Larger osteoclasts, with >5 nuclei per osteoclast, were absent in the controls and exclusively present in all PTH-treated conditions. M-CSF and DC-STAMP expression aligned with osteoclastogenesis. Compared to t = 0 (without PBMC), the RANKL/OPG ratio increased in the second-week exposure group and intermittent groups. IL-1β was upregulated, strongest in the second-week exposure group (p < 0.01). Osteogenic effects were modest and donor-dependent; ALP activity was comparable across groups, though a significant decrease was found between the second week and intermittent regimens. Mineralization varied strongly between donors, with responders and non-responders to PTH treatment. PTH exerts time-dependent effects on osteoclastogenesis in human co-cultures, particularly during early phases. Osteogenic responses varied, suggesting a complex interplay of timing, cell context, and donor variability. These findings support further investigation into optimized timing and delivery of PTH in periodontal regeneration.

## 1 Introduction

Alveolar bone loss is a defining feature of periodontitis, a chronic, multifactorial inflammatory disease driven by microbial dysbiosis and a dysregulated host response (Loos and Van Dyke, 2020). As one of the most prevalent chronic inflammatory conditions globally, severe periodontitis affects nearly 19% of the population, surpassing one billion cases (World Health Organization, 2022), and exhibits parallels with other chronic inflammatory disorders (De Vries et al., 2018; Loos and Van Dyke, 2020; Villoria et al., 2024).

Conventional periodontal therapy aims to halt disease progression but cannot reverse the damage caused by periodontitis, such as loss of alveolar bone, periodontal tissue support, and gingival recession, leading to both functional and aesthetic compromise. Since the 1980s, regenerative approaches targeting the restoration of alveolar bone and periodontal tissues have been investigated using biomaterials, growth factors, and biological agents (Susin and Wikesjö, 2013).

Among these, parathyroid hormone (PTH) and its equally active analog teriparatide (PTH1–34) have shown potential in skeletal and periodontal regeneration. Teriparatide mimics the biological activity of endogenous PTH by binding to its receptor (PTH1R) on osteoblasts, activating cAMP/PKA and PLC/Ca^2+^/PKC signaling pathways, and modulating osteoclastogenesis indirectly through the RANK/RANKL/OPG axis (Jilka, 2007; Sutkeviciute et al., 2019). It also enhances osteoblastic differentiation via RUNX2 (Goltzman, 2008). Parathyroid hormone-related protein (PTHrP), which binds the same receptor, plays vital roles in bone development, tooth eruption, and local signaling in the periodontium (Nagata et al., 2020; Takahashi et al., 2019; McCauley and Martin, 2012). Local PTHrP in particular is upregulated in periodontal ligament fibroblasts during orthodontic tooth movement, resulting in enhanced osteoclast formation at the resorption side (reviewed in Danz and Degen, 2025; Li et al., 2016).

The biological effects of PTH in mice are highly dependent on the delivery mode. Continuous exposure mimics hyperparathyroidism, promoting bone resorption and suppressing osteoblast function (Bilezikian et al., 2009; Wang et al., 2005), whereas intermittent administration enhances bone formation in humans, especially in trabecular bone (Reid, 2015; Neer et al., 2001). Teriparatide, administered systemically in humans at 20 μg/day, has demonstrated safety and efficacy in multiple phase III trials, with site-specific anabolic effects on trabecular microarchitecture and no confirmed link to osteosarcoma or osteonecrosis of the jaw (Kim et al., 2014; Sim et al., 2020).

In periodontal research, PTH has shown potential to stimulate human PDLF cell proliferation, reduce apoptotic signaling in PDL cells, and modulate osteoclastogenesis—namely, enhanced osteoclast differentiation when mouse RAW264.7 cells were co-cultured with PDLF cells, likely due to a decreased OPG/RANKL ratio (Lossdörfer et al., 2006). Preclinical models demonstrate improved bone healing and reduced inflammation with PTH(1–34) therapy (for review, Stutz et al., 2020). Notably, a randomized clinical trial in periodontitis patients showed significant gains in bone height and clinical attachment levels following adjunctive teriparatide treatment (Bashutski et al., 2010).

The periodontal ligament fibroblast (PDLF) model offers a unique platform to study both osteoclastogenesis and osteogenesis, as these cells contribute to both processes and reflect the complex biology of the periodontium (De Vries et al., 2018). In co-culture with PBMCs, PDLFs can support osteoclast differentiation by regulating RANKL and OPG expression. Under physiological conditions, PDLFs inhibit osteoclastogenesis through dominant OPG expression, but in response to inflammatory or hormonal cues, they promote osteoclast precursor recruitment and fusion (Sokos et al., 2015). PDLFs respond directly to PTH signaling by slightly modulating the OPG/RANKL ratio, thereby influencing the differentiation of osteoclast precursors into osteoclasts. This than led to increased osteoclast formation, as revealed by human PDLF–mouse RAW264.7 co-cultures. Osteoclasts formed from human peripheral blood mononuclear cells (PBMCs) cultured with RANKL and M-CSF express functional PTH receptors (Dempster et al., 2005). The co-culture model combining human PDLF cells and human PBMCs therefore integrates these interactions effectively and offers a physiologically relevant *in vitro* platform to investigate the effects of teriparatide on osteoclast formation. A mouse study has demonstrated that stem cells within the periodontal ligament contribute directly to bone formation, since progeny of these cells was found in osteocytes (Ren et al., 2015). In osteodontic studies, however, PDLFs may contribute by activating osteoblasts at the tension site (Danz and Degen, 2025).

Since parathyroid hormone (PTH) can exert both anabolic and catabolic effects on bone, the timing and mode of its stimulation can play a pivotal role in dictating cellular responses. However, temporal insights into PTH-mediated effects remain limited. Despite its clinical relevance, little is known about the temporal effects of PTH on human PDLF-based osteoclastogenesis and osteogenesis. Existing studies have focused on continuous or single-mode delivery, often in non-human models (Ishizuya et al., 1997; Houston et al., 2023). Given the distinct cellular timelines for osteoclast precursor adhesion, migration, and fusion, and the time needed for mineralization in osteogenesis, understanding how the timing of PTH exposure affects these processes is essential.

Therefore, this study aims to investigate how different PTH regimens—continuous, intermittent, and week-specific exposures—affect osteoclast and osteoblast behavior using a human PDLF and PBMC co-culture model. We hypothesize that the timing of PTH stimulation will distinctly modulate osteoclast and osteoblast formation in these human-derived cell types. Given the effect of PTH on RANKL production, especially the earlier timepoint, the first 1/3 of the culture period, and the long exposure of the entire period are likely to have the greatest positive effect on osteoclast formation.

## 2 Materials and methods

### 2.1 Study design and cell cultures

PDLF were derived from extracted third molars that had no signs of inflammation. The middle third of the root was scraped to isolate tissue fragments, from which cells were cultured up to passage three and cryopreserved in liquid nitrogen for future use. Recently, we have shown that these cells express various stem cell markers for mesenchymal stem cells, such as CD73, vimentin, periostin, PLAP-1, Scleraxis and FAPα (Loo-Kirana et al., 2025). In line with international nomenclature, we name them periodontal ligament fibroblasts, but they do possess stem cell characteristics that tenable them to differentiate into mineral depositing cells. For all experiments, cells at passage five were used. Peripheral blood mononuclear cells (PBMCs) were obtained from buffy coats from the local blood bank (Sanquin, Amsterdam, the Netherlands). Permission from Sanquin was provided to use blood products for the purpose of studying bone-cell research related work. Written informed consent was received to use the blood products for this purpose, researchers were not able to trace identity of the donors. Permission for the experiments described here was obtained from the ethical committee of ACTA, under number: 2021-42528.

### 2.2 Titration experiment

PDLF were seeded in 48-well plates at densities of 1.5 × 10^4^ cells/well for osteoclastogenesis and 3 × 10^4^ cells/well for osteogenesis. Before seeding, PDLF were detached from culture flasks using trypsinization. On day 0, the medium was replaced with either standard or mineralization medium (containing 50 μg/mL ascorbic acid and 10 nM β-glycerophosphate, both from Sigma-Aldrich). PBMCs, seeded at 5 × 10^5^ cells/well, were used as osteoclast precursors. The short, equally bioactive derivative of PTH, Teriparatide, containing amino acids 1–34 was used in all experiments, named PTH throughout. To determine the most effective concentration of Teriparatide (Sigma-Aldrich, St. Louis, MO, United States) for osteoclastogenesis, PDLF-PBMC cocultures were exposed to PTH at concentrations of 10^–12^ M, 10^–11^ M, 10^–10^ and 10^–9^ M. In parallel, PDLF cultures were stimulated with mineralization medium with the same range of teriparatide.

### 2.3 Osteogenesis and osteoclastogenesis assays with different exposures of PTH

**FIGURE 1:**
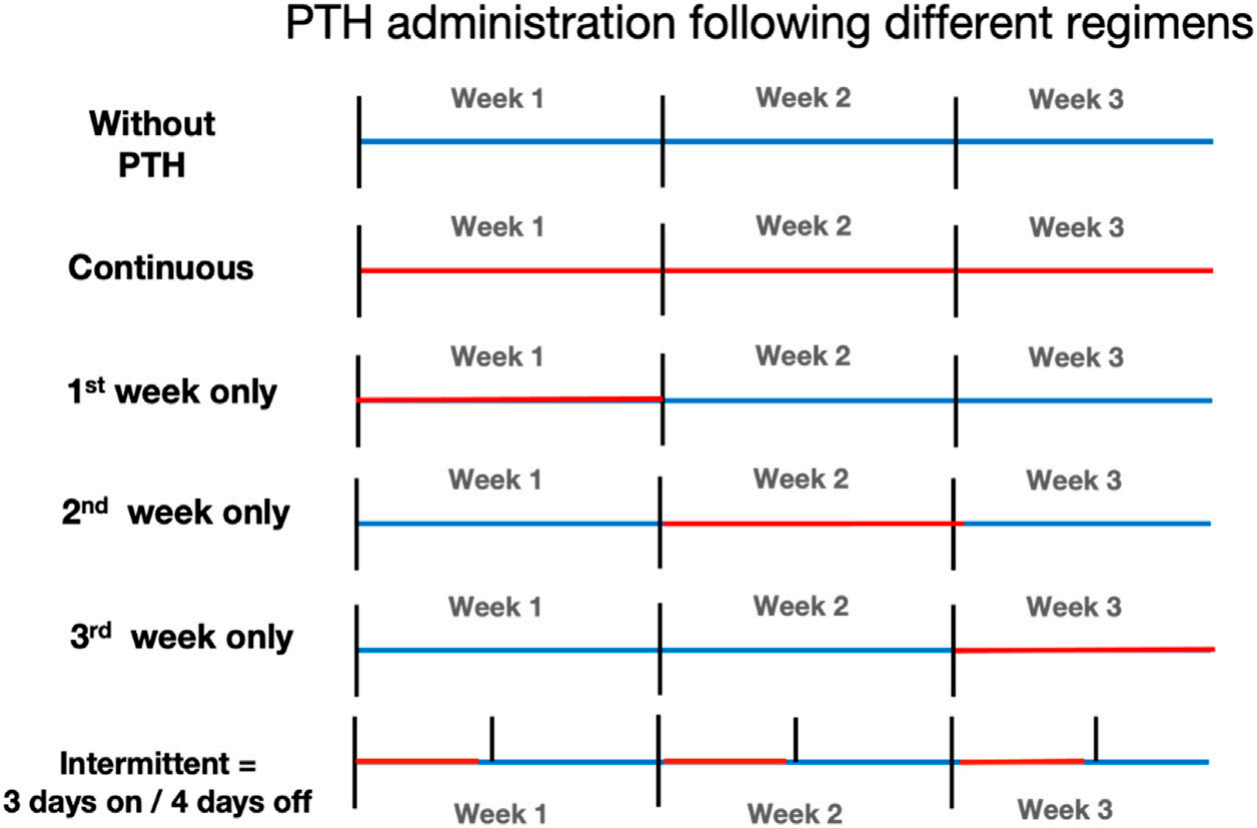
Overview of parathyroid hormone (PTH) administration regimens. Schematic representation of the six PTH exposure protocols used in this study. All cultures lasted 21 days. Regimens included: no PTH (control), continuous exposure (days 0–21), and limited exposure during only the 1st, 2nd, or 3rd week. The intermittent group received PTH every third day of the week (3 days on, 4 days off). Blue bars indicate periods without PTH; Red bars indicate PTH exposure.

To investigate the effect of timing on osteoclast formation and on osteogenesis, PTH was applied at 10^–9^ M either continuous, or only at the first, the second or the third week or intermittently (added once a week during the second refresh step and then washed away). These six schedules are shown in Figure 1. Media were replenished two times a week.

### 2.4 Quantitative polymerase chain reaction (qPCR)

Total RNA was isolated on days 0 and day 21 using the RNeasy Mini Kit (Qiagen, Hilden, Germany). RNA was reverse transcribed to cDNA with the MBI Fermentas cDNA synthesis Kit (Vilnius, Lithuania) using both the Oligo (dT) 18 and D(N)6 primers. qPCR primers were designed for several genes (Table 1). PCR was performed on the LC480 light cycler (Roche, Basel, Switzerland). Hypoxanthine phosphoribosyl transferase 1 (HPRT1) was used as the housekeeping gene for the osteoclastogenesis and osteoclast marker PCRs. The included osteoclastogenesis markers were RANKL, OPG, RANKL/OPG, IL-1β, IL-6. As osteoclast markers, PCRs for M-CSF, DC-STAMP, and TRAcP were used. Gene expression was normalized for HPRT1 expression following the comparative threshold (Ct) method. ΔCt (Ct gene of interest—Ct housekeeping gene) was calculated and relative expression of the genes was determined as 2^−ΔΔct^.

**TABLE 1:** Primer sequences of quantitative real-time PCR analysis.

Gene	Primer sequence (5′ to 3′)	Amplicon length (bp)	Ensembl gene ID
ID β2 microglobulin	Forward: AAg​ATT​CAg​gTT​TAC​TCA​CgT​C reverse: TgA​TgC​TgC​TTA​CAT​gTC​TCg	293	ENSG00000166710
IL-1β	Forward: CTT​TgA​AgC​TgA​Tgg​CCC​TAA​A reverse: AgT​ggT​ggT​Cgg​AgA​TTC​gT	100	ENSG00000125538
RANKL	Forward: CAT​CCC​ATC​Tgg​TTC​CCA​TAA reverse: gCCCAACCCCgATCATg	60	ENSG00000120659
OPG	Forward: CTgCgCgCTCgTgTTTC reverse: ACA​gCT​gAT​gAg​Agg​TTT​CTT​CgT	100	ENSG00000164761
DC-STAMP	Forward: ATT​TTC​TCA​gTg​AgC​AAg​CAg​TTT​C reverse: AgA​ATC​ATg​gAT​AAT​ATC​TTg​AgT​TCC​TT	101	ENSG0000016493
TRAcP	Forward: CAC​AAT​CTg​CAg​TAC​CTg​CAA​gAT reverse: CCC​ATA​gTg​gAA​gCg​CAg​ATA	128	ENSG00000102575
M-CSF	CACCATGCGCTTCAGAGAT CCAGTCCTTGTCAAGGAGAT	208	ENSG00000184371
IL6	ACAGCCACTCACCTCTTCA ACCAGGCAAGTCTCCTCAT	207	ENSG00000136244

### 2.5 TRAcP staining and osteoclast Quantification

After 21 days of culture, cells were fixed with 4% paraformaldehyde and stained for tartrate-resistant acid phosphatase (TRAcP) using the Leukocyte Acid Phosphatase Kit (Sigma-Aldrich). Nuclei were counterstained with DAPI (Invitrogen, Carlsbad, CA, United States). Multinucleated osteoclasts (≥3 nuclei) were counted on micrographs taken from 5 pre-set, fixed, locations per well. Osteoclasts were classified into two categories (3–5 nuclei and >5 nuclei) and counted in duplicate wells. The average values per PDLF donor were used for statistical analysis.

### 2.6 Mineralization assessment

To assess the effect of PTH on mineralization, cells were cultured in the presence of mineralization medium (containing 50 μg/mL ascorbic acid and 10 nM β-glycerophosphate, both from Sigma-Aldrich). Alkaline phosphatase was measured on days 0 and 21 using a previously used protocol (Prins et al., 2024; Steemers et al., 2025). Alkaline phosphatase activity was expressed per cell after determining DNA values in the same supernatant using the Cyquant cell proliferation assay kit (Molecular Probes, Leiden, the Netherlands). Mineral deposition was evaluated using Alizarin Red staining (Sigma-Aldrich). Fixed cells were stained with 2% Alizarin Red solution (pH 4.2) and imaged using a scanner. Previously, we showed that the Alizarin Red staining correlated with calcium deposition (Ceylan et al., 2024) and with expression of osteogenic markers RUNX2, Collagen-I, Osteonectin and Alkaline Phosphatase gene expression (De Vries et al., 2018; Karlis et al., 2020; Loo-Kirana et al., 2025; Ceylan et al., 2024). Mineral deposition was also demonstrated by scanning electron microscopy only in conditions where Alizarin Red was found and mainly extracellular matrix-bound (Prins et al., 2024; Ceylan et al., 2024). Although all of these markers are associated with osteoid, one should refrain from such a term, since this is by definition a non-calcified layer between cuboidal osteoblasts and bone. To the best of our knowledge, true osteoid cell biological assays do not exist.

### 2.7 Statistical analysis

Data were analyzed using GraphPad Prism 9 (GraphPad Software, San Diego, CA, United States).

With the exception of the titration experiment (3 donors), all experimental settings were with periodontal ligament fibroblasts from 7 individual donors. Outcomes were tested for normality. Since all differences were not normally distributed, differences between groups were evaluated using a repeated measures one-way ANOVA using the non-normality Friedman’s post-hoc test, with Dunn’s comparisons of all columns. p-values <0.05 were considered significant. For the titration experiment, 3 donors were used. Graphpad Prism (Boston, MA, United States) software (version 8.0) was used for all graphs and analyses. Linear regression test was used for testing a dose dependent increase in osteoclast formation in the titration test.

## 3 Results

The first experiments aimed to establish the effect of various concentrations of PTH both on osteoclast formation and on osteogenesis. These experiments were performed to determine the optimal concentration for subsequent experiments.

### 3.1 Increasing concentrations of PTH enhance osteoclast formation

**FIGURE 2:**
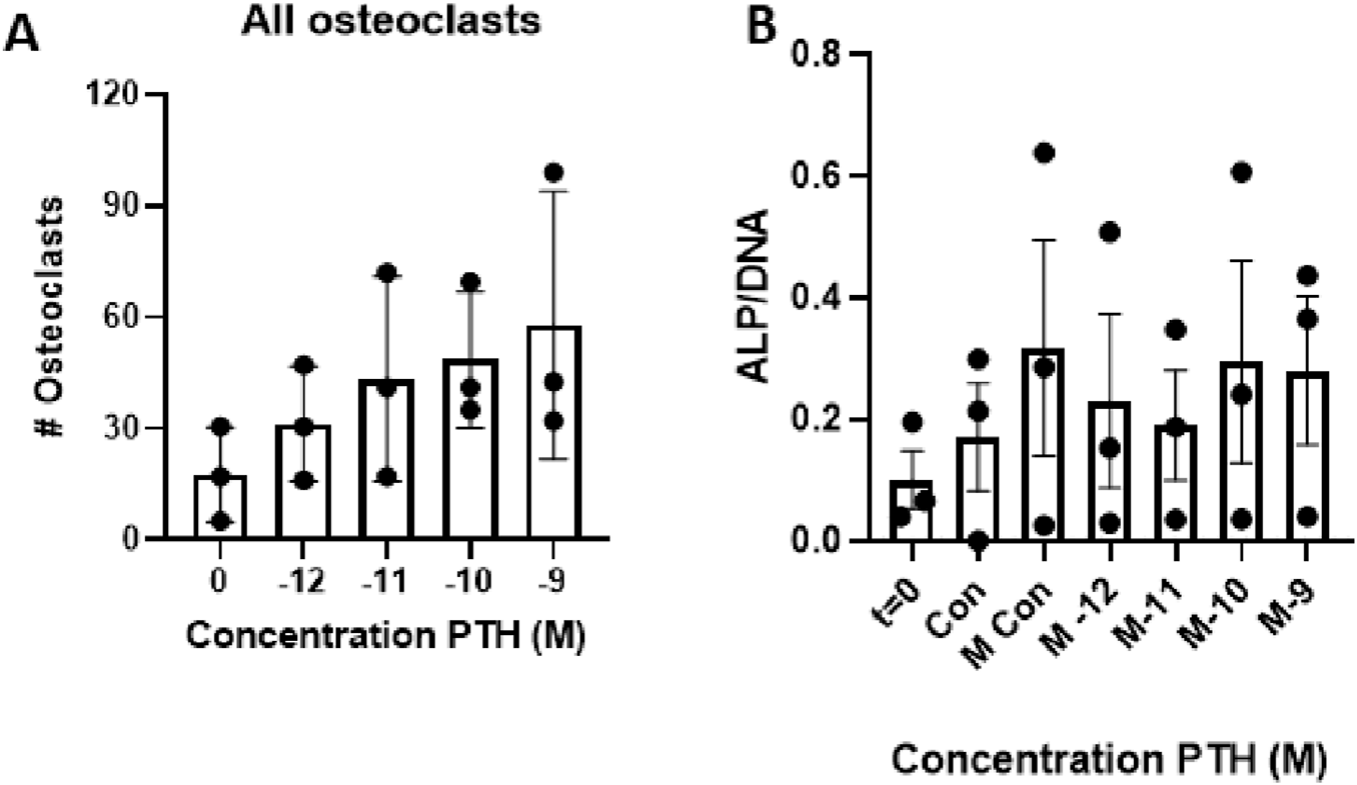
Effect of increasing parathyroid hormone (PTH) concentrations on osteoclast formation and cellular ALP activity. **(A)** Periodontal ligament fibroblasts (PDLFs) were co-cultured with peripheral blood mononuclear cells (PBMCs) for 21 days in the presence of increasing concentrations of teriparatide (10^–12^ to 10^–9^ M). Osteoclast number increased in a dose-dependent manner, with the highest number observed at 10^–9^ M PTH. This concentration was used in subsequent experiments. (n = 3 donors; Linear regression, p = 0.008). **(B)** Alkaline phosphatase (ALP) activity after 21 days of culture with increasing concentrations of PTH (10^–12^ to 10^–9^ M) under osteogenic conditions. PDLFs from three donors (n = 3) were cultured in mineralization medium with or without PTH. ALP activity per cell increased compared to baseline (day 0), but no significant differences were observed between the various PTH concentrations or compared to the mineralization control.

The effect of increasing concentrations of parathyroid hormone (PTH; 10^–12^ M to 10^–9^ M) on the number of osteoclasts formed was assessed. Compared to control conditions (Con), osteoclast formation increased in a concentration-dependent manner (Figure 2A). Linear regression analysis showed a significant concentration dependency (p = 0.008). Based on these findings, the concentration of 10^–9^ M PTH was selected for further experiments.

### 3.2 Increased dosage of PTH does not affect osteogenesis

Three PDLF donors were used to establish the effect of PTH on osteogenesis. As a proxy for osteogenesis, ALP activity was measured and compared between the control group, mineralization group, and the different concentrations of PTH (10^–12^ M to 10^–9^ M) with mineralization medium. Compared to day 0, there seemed to be an increase in ALP activity per cell, but there was no difference between the various concentrations of PTH. Though only three donors were tested, there were no detrimental effects observed in the 10^–9^ M group; thus, this concentration was selected for further experiments (Figure 2B)

### 3.3 Early and mid-term PTH exposure enhances osteoclast number in PDLF-PBMC co-cultures

**FIGURE 3:**
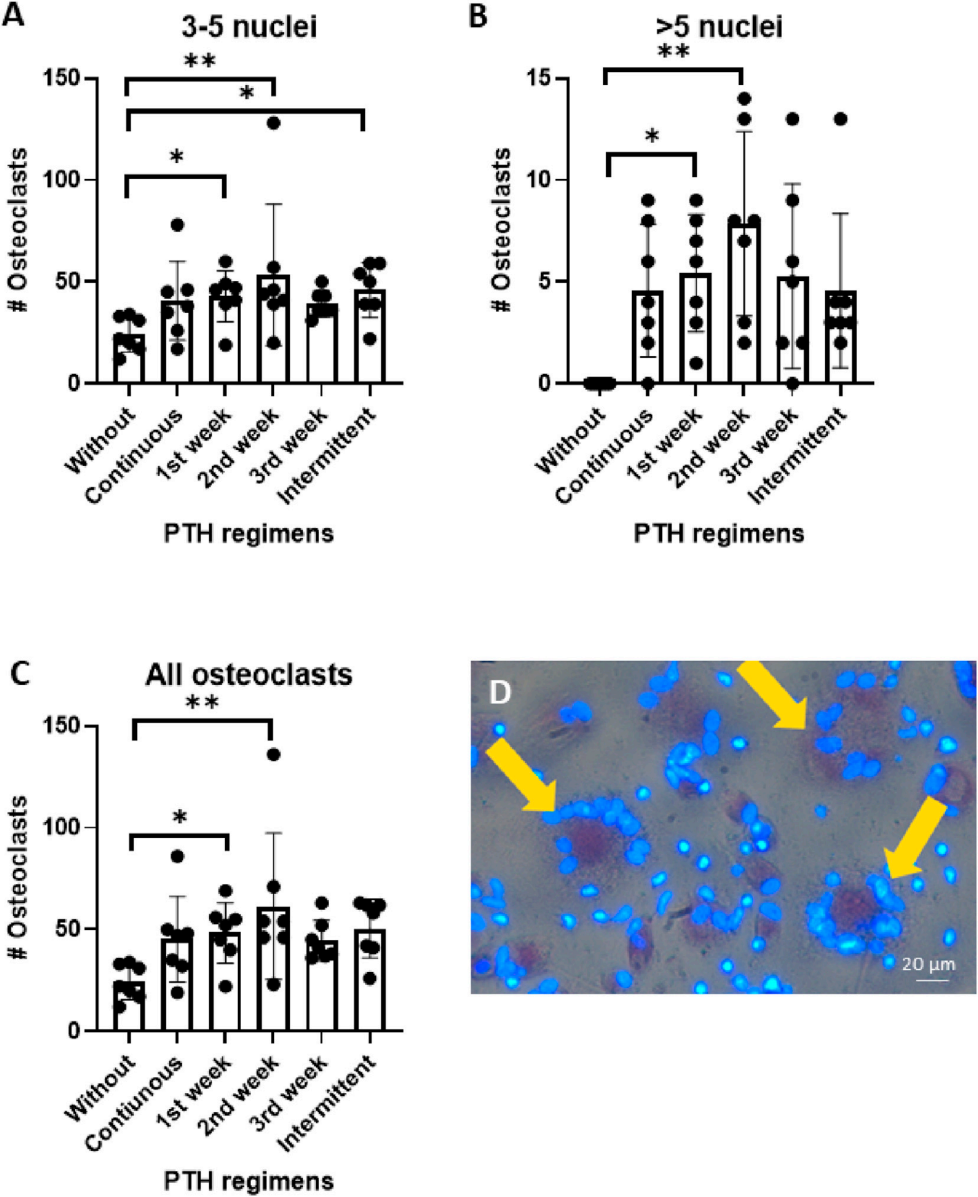
**(A–D)**: Osteoclast formation in PDLF-PBMC co-cultures after 21 of PTH exposure under six different regiments. Cells were cultured without PTH, continuous, 1^st^ week only, 2^nd^ week only, 3rd week only and intermittent. **(A)** Number of osteoclasts with 3-5 nuclei, **(B)** Number of osteoclasts with >5 nuclei, and **(C)** total number of osteoclasts. **(D)** Representative micrograph showing TRAcP-positive multinucleated cells (yellow arrows). Data from 7 donors, *: p < 0.05; **:p < 0.01.

The effect of various PTH regimens on osteoclast formation was assessed after 21 days of co-culture between human periodontal ligament fibroblasts (PDLF) and human peripheral blood mononuclear cells (PBMCs). Cytoplasmic staining with tartrate-resistant acid phosphatase (TRAcP) and nuclear staining with DAPI were used to identify and quantify osteoclasts. Data from seven PDLF donors are shown in Figure 3.

Addition of PTH during the early phases of culture strongly promoted osteoclastogenesis. When specifically addressing the smaller osteoclasts that contained 3–5 nuclei, there were more of those when PTH was administered during the first or second week or intermittently, compared to control treatments. In contrast, continuous PTH exposure, as well as administration during the third week did not result in a change in smaller osteoclast numbers compared to the control (Figure 3A).

Large osteoclasts (>5 nuclei) were not observed in the control group. In comparison, PTH exposure during the first and the second week significantly enhanced the formation of these large multinucleated cells (*p < 0.05, **p < 0.01). The first week and intermittent regimens also induced large osteoclasts, though to a lesser extent (Figure 3B).

Similar to the finding of the two categories of osteoclast size (Figures 3A,B), the total number osteoclasts was especially higher when PTH was added for the first or the second week of culture (Figure 3C). Figure 3D shows the larger osteoclasts that were found after priming with PTH.

These findings demonstrate that early (week 1) and mid-term (week 2) addition of PTH increases osteoclast numbers. Interestingly, continuous culture with PTH did not affect osteoclast numbers.

### 3.4 PTH increases M-CSF expression, no effect on RANKL or OPG expression

To explore the influence of PTH on genes involved in osteoclastogenesis, M-CSF, RANKL and OPG mRNA levels were quantified following 21 days of co-culture. Data from seven PDLF donors were analyzed.

**FIGURE 4:**
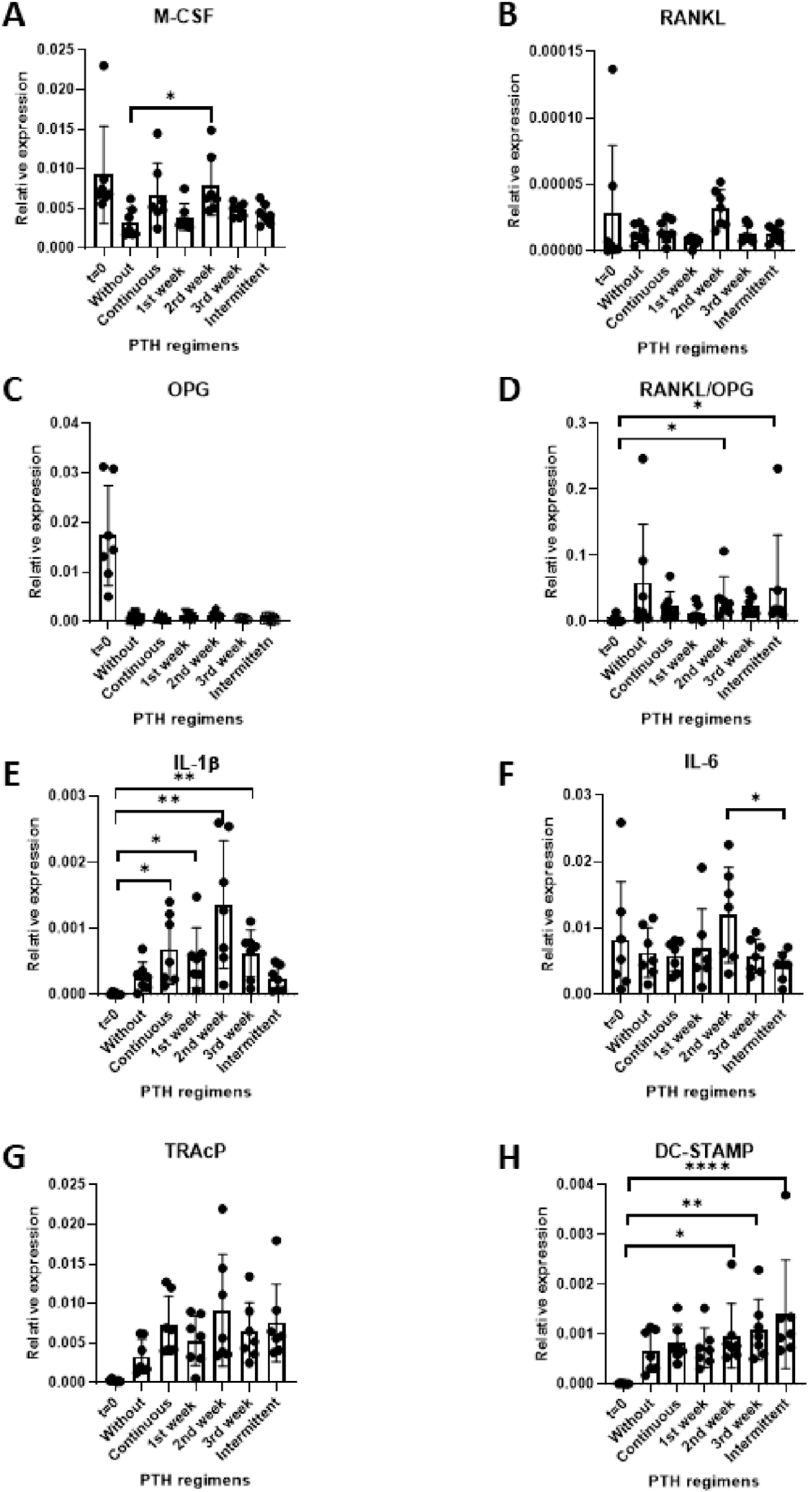
**(A–H)** Gene expression in PDLF (at day 0) and PDLF-PBMC cocultures (at day 21) of culture under different PTH regimes control (no PTH), continuous, 1^st^ week only, 2^nd^ week only, 3^rd^ week only and intermittent. Expression levels of M-CSF **(A)**, RANKL **(B)**, OPG **(C)**, RANKL/OPG ratio **(D)**, IL-1β €, IL-6 **(F)**, DC-STAMP **(G)** and TRAcP **(H)**. Data represent 7 PDLF donors. *p < 0.05; **p < 0.01; ***p < 0.001.

In line with the increased osteoclast formation (Figure 3) M-CSF expression was significantly higher in the group that received PTH during the second week (Figure 4A). RANKL mRNA expression remained relatively low across all conditions and no significant differences between the groups was observed (Figure 4B). OPG expression was initially high at baseline, before PBMCs were added (t = 0), and was lower under all subsequent conditions. This is probably caused by the dilution with OPG-negative PBMCs under co-culture conditions. No significant differences were detected between the various PTH treatment regimens and the control (Figure 4C).

The RANKL/OPG ratio is often used as an indicator of increased osteoclastogenesis. Here, an increase in this ratio was found in the groups treated with PTH during the second week and the intermittent group compared to baseline (*p < 0.05), indicating a shift toward a more pro-osteoclastogenic environment under these conditions (Figure 4D).

Together, these findings indicate that M-CSF and the RANKL/OPG ratio were significantly higher in samples that received PTH during the second week.

### 3.5 PTH upregulates IL-1β and IL-6

To assess the immunomodulatory impact of PTH (10^–9^ M), the expression of the pro-inflammatory cytokines IL-1β and IL-6 was measured at day 21 across different exposure regimens. Expression levels were compared between the conditions as well as between the baseline (t = 0) and control (without PTH), n = 7 donors.

IL-1β expression was significantly upregulated in nearly all PTH treatment conditions compared to baseline. Interestingly, baseline (t = 0), did not differ from the without PTH control and the intermittent condition, whereas all other PTH regimens were significantly higher (Figure 4E).

In contrast, IL-6 expression remained relatively unchanged across most experimental groups. A notable exception was the Intermittent regimen, which was significantly lower than the 2^nd^ week exposure group (Figure 4F).

Overall, these findings suggest that PTH influences pro-inflammatory gene expression.

### 3.6 Unchanged TRAcP gene expression and higher DC-STAMP in some PTH conditions

To assess osteoclast differentiation at the transcriptional level, gene expression of TRAcP (Figure 4G) and DC-STAMP (Figure 4H) were measured by qPCR.

Though an apparent increase of TRAcP expression was observed in all conditions compared to t = 0, none of the statistical analyses were significant.

In contrast, DC-STAMP expression was higher in some of the PTH conditions compared to the t = 0. The 2^nd^ and 3^rd^ week exposure and the intermittent exposure groups were significantly higher (Figure 4H). The 2^nd^ and 3^rd^ week of osteoclast cultures are the typical weeks during which fusion of precursors takes place.

### 3.7 ALP activity remains largely unaffected by PTH exposure across different regimens

**FIGURE 5:**
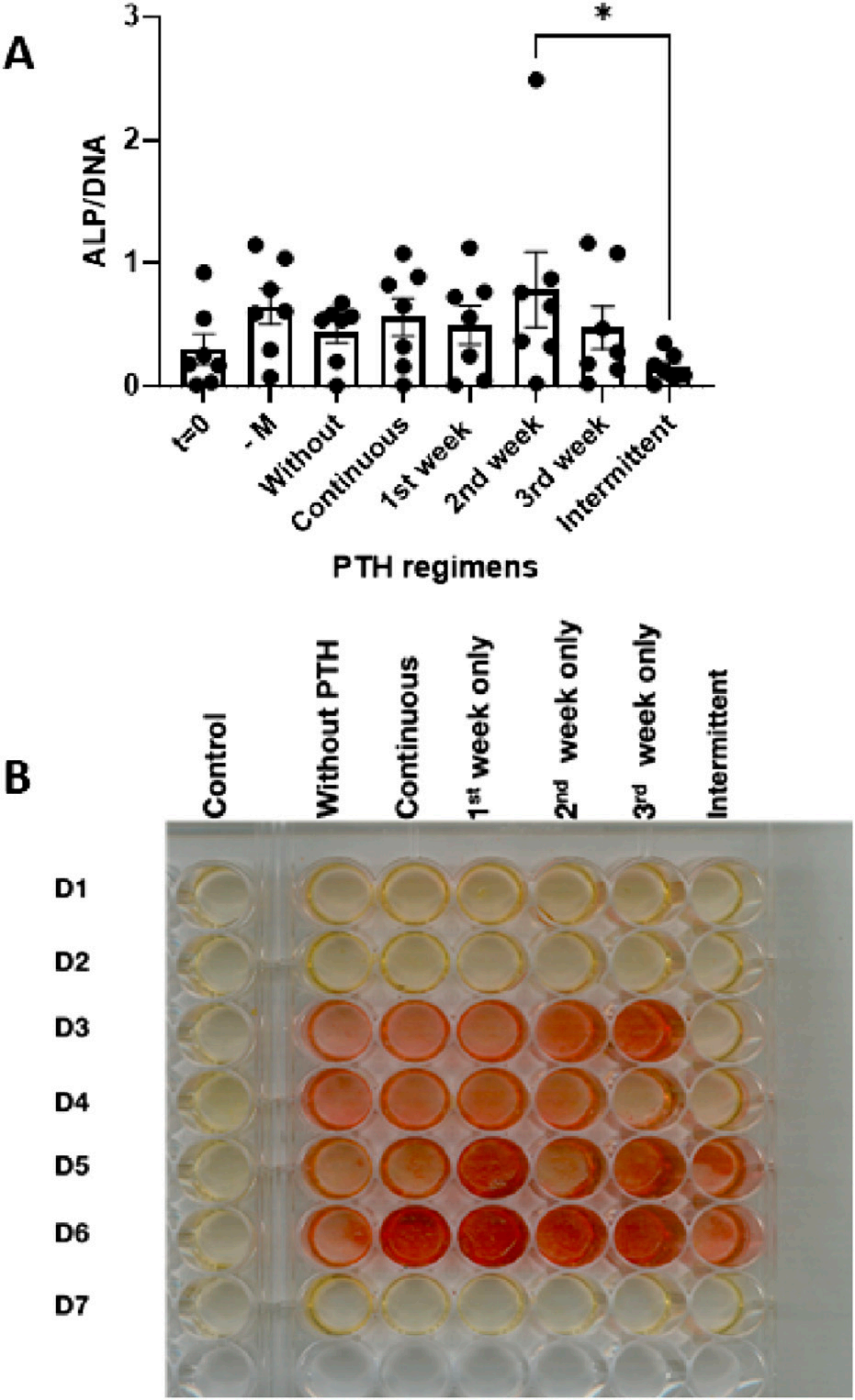
**(A,B)** Osteogenic activity in PDLF cultures assessed after 21 days under osteogenic conditions with various PTH regimens: control (no mineralization), without PTH, continuous, 1^st^ week only, 2^nd^ week only, 3^rd^ week only and intermittent. **(A)** Alkaline phosphatase activity at t = 0 days or at the various conditions at day 14 (n = 7 donors). *: p < 0.05. **(B)** Alizarin red staining showing overall increase with PTH, but with a lot of inter-donor variability.

To evaluate the effect of PTH on osteoblast differentiation, alkaline phosphatase (ALP) activity was measured after 21 days of culture under osteogenic conditions. ALP activity was used as a functional marker of osteogenesis and compared across different PTH exposure regimens. The following conditions were tested: t = 0 (baseline), -M (normal medium, without vitamin C and β-glycerophosphate), Without (osteogenic medium without PTH), and the PTH-treated (continuous, 1st week, 2nd week, 3rd week, and intermittent, Figure 5A).

On average, ALP activity was higher in all conditions at 21 days compared to the day 0 measurement, albeit not significantly. Between the groups, the intermittent was lower than the group that was exposed to PTH during the second week (Figure 5A).

### 3.8 PTH influences mineral deposition as assessed by alizarin red staining

To assess the effect of PTH on mineral deposition, Alizarin Red staining was performed after 21 days of osteogenic culture. This assay visualizes calcium phosphate deposition as an indicator of extracellular matrix mineralization. The tested conditions included five PTH exposure regimens—Continuous, 1st week, 2nd week, 3rd week, and Intermittent—compared against a control group without PTH. Rows in Figure 5B represent individual donors (D1–D7).

Heterogenic responses were noted in this assay. For instance, no visible mineral deposition was observed in donors D1, D2, and D7 (Figure 5B). Across the remaining donors, minimal staining was observed in the control group (Without PTH). Especially donor D6 responded with increased mineralization for nearly all PTH regimens. For all responsive donors, the intermittent regimen showed weaker, patchier or no staining (Figure 5B).

These results indicate that the 7 donors respond heterogeneously to mineralization induction and to PTH treatment.

## 4 Discussion

This study investigated the effect of different timing regimens of parathyroid hormone (PTH) on both osteogenesis and osteoclastogenesis in models using human periodontal ligament fibroblasts (PDLF). Early and mid-phase PTH administration promoted osteoclastogenesis, particularly the formation of larger multinucleated osteoclasts, while continuous or late exposure failed to elicit a similar response. Osteogenesis outcomes, conversely, were donor-dependent and less consistently affected by PTH as assessed by ALP activity and Alizarin Red staining. The study is the first, using this model system, to demonstrate the temporal effects of PTH administration on osteogenesis and osteoclastogenesis.

Our titration experiments did not show dose-dependent effects of PTH stimulation on osteogenesis. This is in contrast to one of the early *in vivo* studies (Isogai et al., 1996) who observed a significant dose-dependent effect on osteoblast-like cells that were isolated from mouse calvaria as measured by alkaline phosphatase (ALP) when using higher PTH concentrations between approximately (∼10^–11^ to 10^–8^ M).

PTH caused a dose dependent increase of the number of osteoclasts. The highest PTH dose 10^–9^ M yielded the highest number of osteoclasts after 3 weeks of co-culture of PDLF and PBMCs. This is an interesting finding, because most studies that investigated molecular and cellular mechanisms of PTH have mainly focused on osteoblast lineage cells only (Jacome-Galarza et al., 2011). Our co-culture cell system allowed for investigation of PTH effects on both osteogenesis and osteoclastogenesis.

Lossdörfer et al., 2011 used a co-culture system of PDLF with murine RAW 264.7 cells to investigate the effects of PTH stimulation on osteoclast formation. However, here, osteoclastogenesis was further stimulated by adding M-CSF and RANKL. Therefore, as they mentioned as a limitation of that study, osteoclastic differentiation cannot be attributed to a direct effect of PTH. Our study put the capacity of PDLF to orchestrate osteoclast formation to the forefront, without any addition of cytokines. In other words, the PTH effects were due to the combined PTH response of both PFLFs and PBMC. Notably, the *in vitro* concentration of 10^–9^ M is higher than physiological circulating levels in humans after subcutaneous injection of 20 µg. In humans, teriparatide reaches peak serum concentrations of ∼60 pg/mL (equivalent to ∼10^–11^ M) within 30 min and has a half-life of ∼1 h (Satterwhite et al., 2010). However, it is conceivable that PTH concentrations are much higher around target cells, such as osteoblasts and PDLF. Therefore, *in vitro* cultures often use higher concentrations.

Regarding the PTH stability of Teraparatide *in vitro*, it was shown by (Rickard et al., 2006) that a PTH concentration of 10 nM remained stable up to 72 h when added with human mesenchymal stem cell cultures, indicating that the regimens used in the present study had active PTH for at least 72 h.

Importantly, PTH enhanced osteoclastogenesis when added during the first and second week. This effect was evident not only in total osteoclast numbers that were assessed, but particularly in the formation of large multinucleated osteoclasts (>5 nuclei), where significantly more of these larger cells were found in the early and mid-phase PTH groups. This suggests that PTH acts as a priming factor for early osteoclast fusion events. The higher expression of M-CSF in the culture regimen of adding PTH in the second week could shed light on the mechanism behind the increased osteoclast formation under this circumstance. Our findings suggest that time-specific PTH exposure enhances osteoclastogenesis by upregulating M-CSF signaling.

In contrast to M-CSF, RANKL expression remained low across all groups and was not affected by PTH exposure. These findings diverge from those of another PDLF co-culture study, where a 24-h intermittent PTH stimulation significantly upregulated RANKL gene expression (Lossdörfer et al., 2011). In addition, we did not observe the upregulatory effect of continuous PTH administration on RANKL expression reported by Ma et al. (2001), although it is important to note that their findings were based on an *in vivo* animal model. Inevitably, we were not able to study RANKL expression at early time-points only, since all regiments were included in the analysis. Therefore, we could only compare gene expression at the end-point of 21 days. We cannot exclude that PTH could have primary effects on gene expression at earlier time points, which could be indicated as a shortcoming of this study. The findings by Lossdörfer et al. (2011) indicate that PTH particularly modifies gene expression also at early stages. The present study, however, focused on longer duration effects, after exposures of a week, continuous or intermittently.

The RANKL/OPG ratio is a widely accepted indicator of osteoclastogenic potential, with higher values reflecting a shift toward enhanced osteoclast differentiation. In our study, we observed a significant increase in the RANKL/OPG ratio in the groups treated with PTH during the second week and under intermittent exposure compared to baseline, suggesting a more pro-osteoclastogenic environment under these conditions. These findings align partially with those of Lossdörfer et al. (2011), who reported that intermittent PTH (1–34) (here: repeated) stimulation for 24 h per cycle led to a significant reduction in OPG expression alongside an increase in RANKL transcription, ultimately decreasing the OPG/RANKL ratio. While we did not observe upregulation of RANKL alone, the observed shift in the ratio suggests that even modest changes in RANKL or OPG levels may be sufficient to tip the balance toward osteoclastogenesis in this co-culture model.

We also observed that PTH modulated inflammatory cytokine expression. IL-1β was significantly upregulated across most PTH conditions, suggesting that PTH may exert part of its osteoclastogenic effect by creating a pro-inflammatory microenvironment. IL-1β is known to be a potent stimulator of bone resorption and has been shown to synergize with PTH in promoting osteopenia in fetal rat long-bone cultures, even when both are used at very low concentrations (Dewhirst et al., 1987). Interestingly, however, the regulatory relationship may be bidirectional. Lippuner and colleagues (Lippuner et al., 1999) demonstrated that systemic administration of recombinant IL-1β to rats induced a dose-dependent suppression of circulating PTH levels without significantly altering serum calcium. This suggests that IL-1β may exert negative feedback on PTH secretion, while simultaneously promoting bone resorption through osteoclast activation. Taken together, these findings underscore a complex interplay between PTH and IL-1β in bone remodeling, with potential relevance to both inflammatory and hormonal regulation of osteoclastogenesis.

Although early *in vitro* studies reported increased IL-6 production in by stromal/osteoblastic cells in response to PTH (Grey et al., 1999), our study showed IL-6 showed limited changes, with a small but significant decrease in the intermittent group compared to the second-week exposure.

Another relevant finding is that DC-STAMP, a key fusion marker for osteoclasts, was upregulated significantly in the second and third week and intermittent regimens. This aligns with our observed increase in large multinucleated osteoclasts and supports the notion that PTH enhances not only precursor survival (via M-CSF) but also their fusion capacity. TRAcP gene expression, although numerically higher than baseline, did not significantly differ between PTH regimens and controls. This may reflect post-transcriptional regulation or saturation of TRAcP expression during prolonged cultures.

While our experiment refined the knowledge on PTH’s role on specifically the stage preluding osteoclast formation, its impact on osteogenesis was modest and subject to donor heterogeneity. ALP activity measured after 21 days of culture showed no significant differences between treatment groups and controls. This is slightly in contrast with previous findings by Lossdörfer and others (Lossdörfer et al., 2011) who reported that PTH stimulation inhibited ALP activity in confluent PDLF. However, as noted earlier, because of the experimental set-up, our study could only study ALP activity after 21 days, probably not optimal for this marker that is usually measured after 14 days with mineralization medium (Loo-Kirana et al., 2025; Karlis et al., 2020). Similarly, we observed no significant difference between the continuous and intermittent PTH groups. The 21 days time point for ALP could be suboptimal, as it usually peaks at 14 days (De Vries et al., 2018) in periodontal ligament fibroblast cultures. Interestingly, a statistically significant difference was found between the intermittent and second-week-only PTH groups. This suggests that PTH is probably most effective for 7 days halfway the mineralization assays and that intermittent treatment of 3 days versus a break of 4 days is not beneficial for ALP activity. The Alizarin Red staining is in line with the reduced ALP activity, since lower calcium deposits are seen under intermittent conditions. To further fine-tune PTH’s effects on osteogenesis, future studies should study the ALP activity changes due to PTH at more time points, probably at 3, 7, 14, and 21 days, such as in one of our previous studies (De Vries et al., 2018). Such studies should also include gene expression data of genes such as RUNX-2, osteopontin, and osteocalcin, genes that are upregulated in periodontal ligament osteogenesis assays (Karlis et al., 2020; Loo-Kirana et al., 2025).

Although it has long been recognized that periodontal ligament fibroblasts may play a role in osteogenesis, one could argue whether they make part of the bone remodeling regulatory system (BRRS) (Danz and Degen, 2025). In principle, the mere function of the PDL is against bone formation (anti-anklylosis) and against resorption. For the latter, the at least 100-fold overexpression of OPG over RANKL is an interesting scientific argument reviewed by Sokos et al. (2015). Clearly, the role in orthodontic tooth movement has been well established, where it is indispensable in directing both osteoclast formation at the traction site as well as osteoblastic activity at the tensions site. In that context, PTH infusion has shown to increase the speed of tooth movement, by acting on osteoclast formation (Soma et al., 1999). PTH, that recruits osteoblast activity *in vivo*, may have a slightly different effect on periodontal ligament fibroblasts, since it causes a downregulation of sclerostin (Bellido et al., 2005). We have recently shown that periodontal ligament fibroblasts hardly express sclerostin: osteocytes expressed 45000 times more sclerostin (Pigeaud et al., 2023).

Our hypothesis that different PTH administration regimens would distinctly modulate both osteoclast and osteoblast formation is only partially supported by our data: osteoclastogenesis was influenced by timing, whereas osteogenic responses were limited and variable. While variability in responsiveness to teriparatide treatment has been previously documented, with roughly 15% of osteoporosis patients not responding well to the therapy (Heaney and Watson, 2011) the findings of Larraz and colleagues (Larraz-Prieto et al., 2025) may offer a mechanistic explanation for this phenomenon. They identified CXCR4 as a PTH-responsive gene that regulates both osteoclast and osteoblast function. This variability may be attributed to interindividual genetic differences at the CXCR4 locus, which a genome-wide association study (GWAS) linked to differential bone mineral density responses to teriparatide (Alonso et al., 2023). These findings suggest that genetic variation in CXCR4 expression and function may underlie the heterogeneous anabolic response to PTH therapy.

A limitation of the present study is that all outcomes were assessed at a single endpoint (day 21), which restricts insight into the temporal dynamics of cellular events that may be influenced by different parathyroid hormone administration regimens over time. One could also consider the translation to *in vivo*, where primarily a net anabolic effect is seen. Periodontal ligament fibroblasts were studied as a source of osteogenic cells, but *in vivo*, they may be influenced by neighoring osteoblasts and osteocytes, that are more responsive to bone regulators such as sclerostin (Pigeaud et al., 2023). Lack of mechanical loading could be considered as another limitation of the present study. The novelty of the present study is however, that it sheds light on the timing of PTH, which is more pronounced on the osteoclastic aspect of PTH.

Optimization of timing and delivery is relevant for PTH’s application, for instance in dentistry. Recent preclinical work by Latimer and colleagues (Latimer et al., 2025) demonstrated that Abaloparatide (ABL), a next-generation PTH analog of similar structure to Teriparatide PTH (1–34) but with a better anabolic profile, enhances osteogenesis in extraction sockets prior to implant placement and accelerates peri-implant bone formation in the early phase of healing in mice. They speculated that the strategic timing of PTH administration in conjunction with dental treatment may enhance treatment outcomes. Further investigation into the specific window of time during which ABL exerts its greatest regenerative effect may be an interesting and valuable area for future research.

## 5 Conclusion

This study demonstrates that the effects of parathyroid hormone (PTH) on periodontal regeneration are time- and context-dependent. Early and mid-phase exposure to PTH significantly promoted osteoclastogenesis, particularly enhancing the formation of large multinucleated osteoclasts, likely via upregulation of M-CSF and DC-STAMP. In contrast, osteogenic outcomes, assessed by ALP activity and mineral deposition, were modest, variable across donors, and not robustly enhanced by PTH under the tested conditions. The significant difference between intermittent and second-week exposure regimens suggests that timing may play a role in osteoblastic activity. These findings emphasize the importance of optimizing hormone delivery strategies in regenerative approaches. Next-generation bone anabolic agents might be interesting to investigate *in vitro*, particularly to explore the relevance of timing using appropriate human cell systems. Future studies should include temporal gene expression analyses and *in vivo* validation to better define the optimal therapeutic window and agent selection.

## Data Availability

The raw data supporting the conclusions of this article will be made available by the authors, without undue reservation.
